# *Thyreophagus tauricus* sp. n., a New Subcortical Mite Species (Acari: Acaridae), with a COX1 DNA Sequence Analysis of Several Economically Important Species of *Thyreophagus* †

**DOI:** 10.3390/ani13223546

**Published:** 2023-11-16

**Authors:** Pavel B. Klimov, Vasiliy B. Kolesnikov, Vladimir A. Khaustov, Alexander A. Khaustov

**Affiliations:** 1 X-Bio Institute, Tyumen State University, 10 Semakova Str., 625003 Tyumen, Russia; jukoman@yandex.ru (V.B.K.); kh4ustov93@yandex.ru (V.A.K.); alkhaustov@mail.ru (A.A.K.); 2 Lilly Hall of Life Sciences, Purdue University, G-225, 915 W State St., West Lafayette, IN 47907, USA; 3 Federal Public Budgetary Scientific Institution, All-Russian Research Institute of Plant Protection, 396030 Voronezh, Russia

**Keywords:** astigmatid mites, sexual species, morphology, molecular identification, Crimea, laboratory culture

## Abstract

**Simple Summary:**

In recent years, there has been a growing interest in finding sustainable and environmentally friendly solutions to combat agricultural pests while minimizing the adverse impacts of chemical pesticides. Species of the genus *Thyreophagus* have emerged as a valuable asset in this pursuit. These mites are utilized as factitious prey for the mass rearing of predatory mites. Predatory mites, in turn, play a crucial role in biological pest control—they feed on a variety of agricultural pests such as spider mites, thrips and other small arthropods that harm crops. Despite their significance, we do not know much about *Thyreophagus* mites because many species live in hidden habitats and are difficult to study. As part of our survey, we discovered a new species, *Thyreophagus tauricus*, and provide detailed descriptions of its different life stages. Genetic sequencing was also performed to identify this new species and compare it with others: *Thyreophagus corticalis* (broadly distributed Palearcic species), *Th. calusorum*, *Th. entomophagus* (economically important factitious prey mites). We also correct some mistakes in mite identification, particularly the economically important species *Th. entomophagus*, which can be crucial for future studies and biocontrol applications.

**Abstract:**

As part of a survey of the subcortical astigmatic mites of Crimea, we discovered a new sexual acarid species, *Thyreophagus tauricus* sp. n. This species was cultured in the laboratory to correlate the adult and deutonymphal stages. Using specimens obtained by these rearing experiments, we provide a detailed description of *Th. tauricus* (light microscopy, SEM) based on females, males and heteromorphic deutonymphs. Furthermore, to facilitate molecular identification, the entire COX1 gene was also sequenced for this and three other Palearctic species reared in the lab as pure cultures. Adults of *Th. tauricus* sp. n. are distinct among all other species of the genus by the presence of flattened, button-shaped or minute spiniform setae *s* III and IV, which are well-developed spiniform in all other known species of *Thyreophagus*. Heteromorphic deutonymphs of *Th. tauricus* are distinct from all other species of *Thyreophagus* by the presence of well-developed setae *cm* on the dorsal part of the subcapitular remnant (absent all other species). *Th. tauricus* is morphologically very similar to *Th. corticalis*; however, COX1 K2P distances between these two species were large, 19.8%. COX1 K2P distances between *Th. tauricus* and other species (*Th. entomophagus*, *Th.* “*entomophagus*” NC 066986.1, *Th. calusorum*, *Th. corticalis*) ranged between 20.1 and 24.3%. We show that the GenBank sequence of *Th*. “*entomophagus*” from China (NC 066986.1) was probably misidentified.

## 1. Introduction

The genus *Thyreophagus* Rondani, 1874 (Acari: Acaridae) is distributed worldwide, except Antarctica [1,2,3,4]. Various species of *Thyreophagus* occur in subcortical habitats, stored food, in association with scale insects, and nests of wasps and bees [1,2,3,4,5,6,7,8]. Some *Thyreophagus* species are beneficial or economically and medically important [9,10,11,12,13,14]. For example, *Thyreophagus entomophagus* (Laboulbène and Robin, 1862) is widely used as factitious prey for mass rearing of phytoseiid predatory mites for biocontrol applications [9]; additional beneficial species have been recently identified, tested and used in industrial settings, such as *Thyreophagus calusorum* Klimov, Demard, Stinson, Duarte, Wäckers et Vangansbeke, 2022 [15] and *Thyreophagus cracentiseta* Barbosa, OConnor and Moraes, 2016 [7]. In the genus *Thyreophagus*, there are 34 nominal species and one subspecies; however, the actual diversity of the genus *Thyreophagus* may be underappreciated, as most species prefer hidden habitats, and can be easily overlooked [1]. Living in habitats that have a limited number of natural predators is also a key biological feature that makes these mites well suited as factitious prey in industrial settings. Mites of the genus *Thyreophagus*, known for their slow movement and a lack of certain natural defenses (like long setae), are especially useful as prey for mass produced phytoseiid mites.

Most commonly, mite taxonomists collect and describe females, while other taxonomically important stages, males (absent in asexual species) and heteromorphic deutonymphs are omitted. These ontogenetic stages can be obtained through rearing in the lab, but this is rarely performed. As a result, only four species are currently recognizable from both adult and deutonymphal stages: *Th. australis* Clark, 2009, *Th. corticalis* (Michael, 1885), *Th. entomophagus* and *Th. calusorum* [1,16,17,18].

Because of its economic importance and the presence of potentially interesting biological features related to asexuality, a comprehensive study of the *Thyreophagus* species morphological and molecular diversity is needed. Such a study should be based on different stages (females, males, heteromorphic deutonymphs) obtained through rearing experiments of pure cultures and/or through the correlation of ontogenetic stages in the wild populations. Rearing in the lab is also important to confirm if a species is indeed asexual.

As part of a survey of the subcortical astigmatic mites, we discovered a new sexual species, *Thyreophagus tauricus* sp. n., from Crimea. This new species was cultured in the laboratory to correlate the adult and deutonymphal stages. Based on specimens obtained by these rearing experiments, we provide a detailed description (light microscopy, SEM) based on females, males and heteromorphic deutonymphs. To facilitate molecular identification and species delimitation, we sequenced the entire COX1 gene (a DNA barcoding gene) of four Palearctic species, including *Th. tauricus* its closely related species, *Th. corticalis.* We used these and GenBank sequence data to compare the genetic distances among different species of *Thyreophagus*. 

## 2. Materials and Methods

Fallen twigs of different species of deciduous trees were collected, transferred into a laboratory and examined for the mites under a dissecting microscope. Mites were collected using a camel brush and preserved in 96% ethanol, cleared in lactic acid 80% for 1–2 days and mounted in Hoyer’s medium, followed by 7-day drying at 60 °C.

For rearing in the lab, live mite specimens were transferred into rearing units and maintained on a mixture of yeast and bran as a food source. The purity of a culture was confirmed via morphological identification of a large of number of mites (n = 50) harvested from the same culture.

Cultures were established for following species: *Thyreophagus calusorum*—USA: Florida, Fort Pierce, branch on ground, stick2, 12 October 2020, Emilie Demard, 27°25′34.5″ N 80°24′22.7″ W, PBK 20-0101-007); *Thyreophagus entomophagus*—Russia: Tyumenskaya oblast’, Tyumen, culture from a Russian biocontrol company, 20 November 2021, Vladimir Khaustov, PBK 20-0101-199; *Thyreophagus corticalis*—Russia: Voronezhskaya oblast’, Voronezh, mixed forest, under bark of *Acer platanoides*, 14 November 2021, Vasiliy Kolesnikov, PBK 20-0101-061; *Thyreophagus tauricus* sp. n. (see below).

Images were taken from multiple focal planes and assembled in Helicon Focus 7.6.4 Pro (algorithm B, rarely A) with subsequent manual editing (retouching) of misassembled regions. Individual, partially overlapping images were merged into a full panorama in Adobe Photoshop 22.2.0. Line drawings were made in Photoshop 22.2.0 using microphotographs as the background. Background images were taken using a Euromex Color HD-Ultra camera and a Bioptic C-400 (Bioptic, Moscow, Russian Federation) microscope equipped with bright field and differential interference contrast optics (DIC). Publication-quality microphotographs were taken using an Axio Imager A2 (Carl Zeiss, Oberkochen, Germany) compound microscope equipped with DIC and phase contrast optics and an Axiocam 506 color (Carl Zeiss, Oberkochen, Germany) digital camera. For scanning electron microscope imaging, alcohol-preserved mites were dried in a JFD 320 freeze dryer (JEOL, Tokyo, Japan), dusted with gold, and scanned using a JEOL-JSM-6510LV SEM microscope. Specimens used for SEM were not preserved.

In descriptions, idiosomal chaetotaxy follows [19]; the terminology of coxisternal setae follows [20]; for appendages, the chaetotaxy and solenidiotaxy follow Grandjean for palps [21] and legs [22]. Designations of tarsal dorsoapical setae of legs III–IV follow [8]. All measurements are given in micrometers (μm).

For each cultured species, genomic DNA was extracted from 200 females obtained from a pure culture (see above), using a QIAamp DNA Micro kit (Qiagen, Venlo, The Netherlands) with modifications as described here [23]. Illumina sequencing libraries were generated and sequenced commercially on an Illumina NovaSeq 6000 sequencing system. Short Illumina reads were assembled in SPAdes v.3.15.5 [24] as follows: metaspades.py -t 24 -m 240 -1 ${name}_R1_001.fastq -2 ${name}_R2_001.fastq -o ${name}. Full-length COX1 sequences were found using a local NCBI BLAST search and then deposited into the GenBank database, accession IDs: OR640973-OR640976. Genetic distances were calculated in PAUP v.4a168 [25] as follows: begin paup; dset distance = p; savedist format = tabtext undefined = asterisk file = 1_p_distances.tab; dset distance = k2p; savedist format = tabtext undefined = asterisk file = 2_k2p_distances.tab; end.

## 3. Results

### 3.1. Molecular Identification

For the COX1 gene of *Th. tauricus*, the top *blastx* hit (translated nucleotide to protein analysis, genetic code = invertebrate mitochondrial) was *Thyreophagus entomophagus* from China (NC_066986.1), with a 90.31% amino acid sequence similarity. Our sequence of *Th. tauricus* was therefore classified in the genus *Thyreophagus* correctly. However, the *Th. entomophagus* GenBank entry did not match our sequence of *Th. entomophagus*, with K2P COX1 nucleotide distance = 0.212 (21.2%) (Table 1). Since our sequence was obtained from specimens from a pure culture and given the careful identification of our morphological co-vouchers (Fain, 1982), we believe that the GenBank sequence of *Th. entomophagus* (NC_066986.1) may be misidentified.

Given our dataset, *Th. tauricus* has the closets match to *Th. corticalis*, COX1 K2P nucleotide distance = 0.198 (19.8%) vs. 0.201–0.243 for other species (Table 1). This result makes sense because these two species have only minor morphological differences (see below).

### 3.2. Morphological Description

#### 3.2.1. Genus *Thyreophagus* Rondani, 1874

*Thyreophagus* Rondani, 1874: 67 (=*Moneziella* Berlese, 1897; *Monetiella* Berlese, 1897; *Monieziella* Berlese, 1897; *Fumouzea* Zachvatkin, 1953; *Michaelopus* Fain and Johnston, 1974).

Type species *Thyreophagus entomophagus* (Laboulbène and Robin, 1862) (=*Acarus entomophaus* Laboulbène, 1852 (nomen nudum)), by monotypy.

#### 3.2.2. *Thyreophagus tauricus* sp. n.

Description

**Figure 1:**
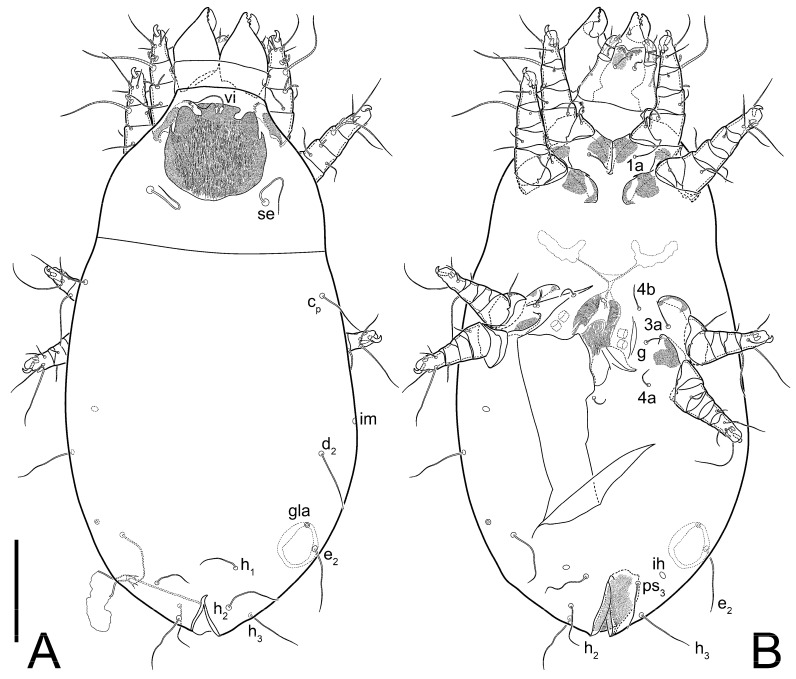
*Thyreophagus tauricus* sp. n., female, holotype: (**A**)—dorsal view; (**B**)—ventral view. Scale bar 100 µm.

**Figure 2:**
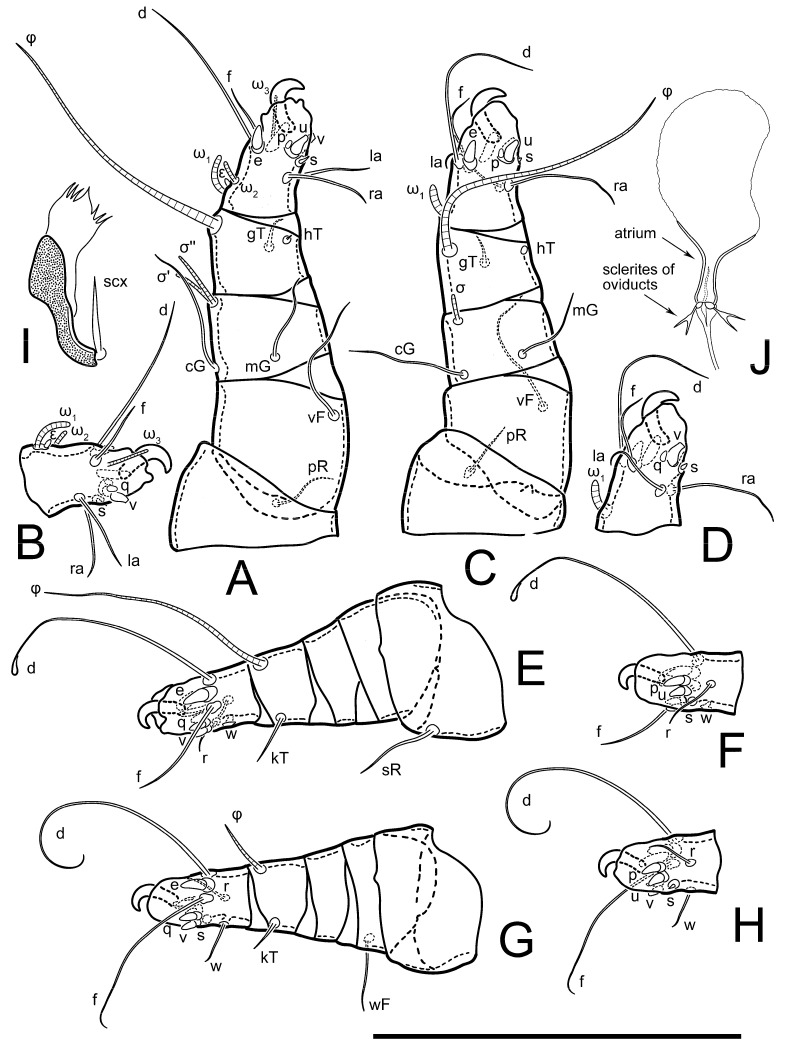
*Thyreophagus tauricus* sp. n., female, paratype: (**A**)—right leg I, posterior view; (**B**)—tarsus I, anterior view; (**C**)—right leg II, posterior view; (**D**)—tarsus II, anterior view; (**E**)—right leg III, anterior view; (**F**)—tarsus III, posterior view; (**G**)—right leg IV, anterior view; (**H**)—tarsus IV, posterior view; (**I**)—supracoxal sclerite and Grandjean’s organ; (**J**)—spermatheca. Scale bar 100 µm.

**Figure 3:**
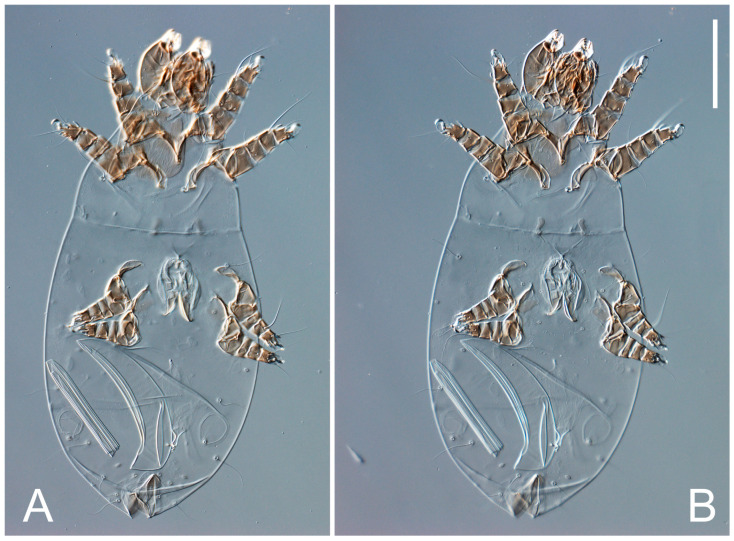
*Thyreophagus tauricus* sp. n., female, paratypes, DIC images: (**A**)—dorsal views; (**B**)—ventral views. Scale bar 100 µm.

**Figure 4:**
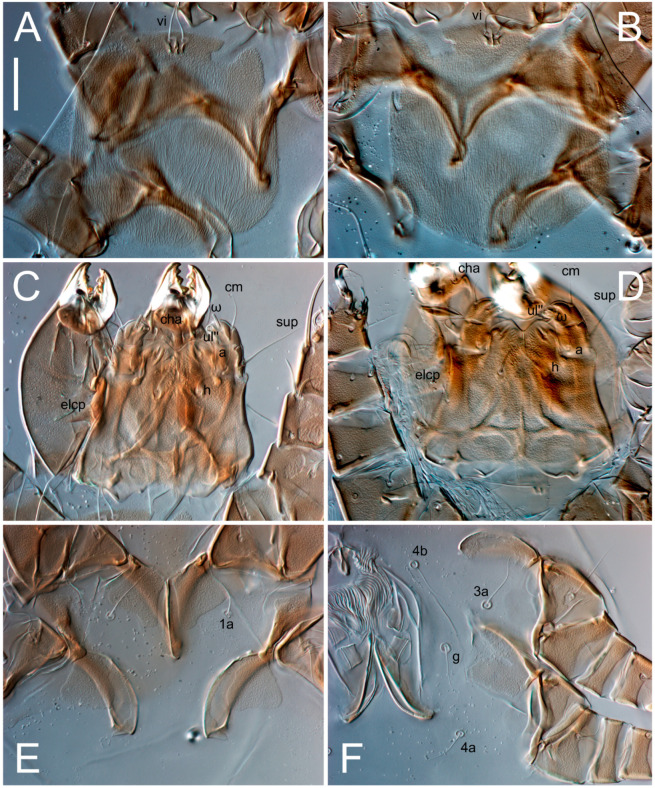
*Thyreophagus tauricus* sp. n., female, paratypes, DIC images: (**A**,**B**)—prodorsal shields; (**C**,**D**)—gnathosoma, ventral views; (**E**)—coxisternal fields I–II; (**F**)—coxisternal fields III–IV. Scale bar 20 µm.

**Figure 5:**
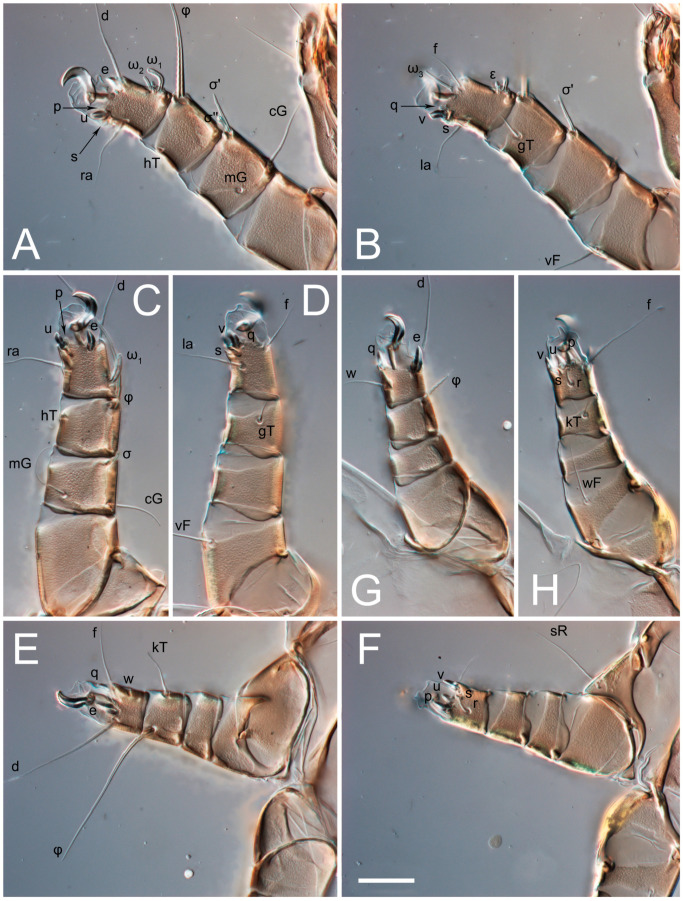
*Thyreophagus tauricus* sp. n., female, paratypes, DIC images: (**A**)—left leg I, posterior view; (**B**)—left leg I, anterior view; (**C**)—left leg II, posterior view; (**D**)—left leg II, anterior view; (**E**)—left leg III, anterior view; (**F**)—left leg III, posterior view; (**G**)—left leg IV, posterior view; (**H**)—left leg IV, anterior view. Scale bar 20 µm.

**Figure 6:**
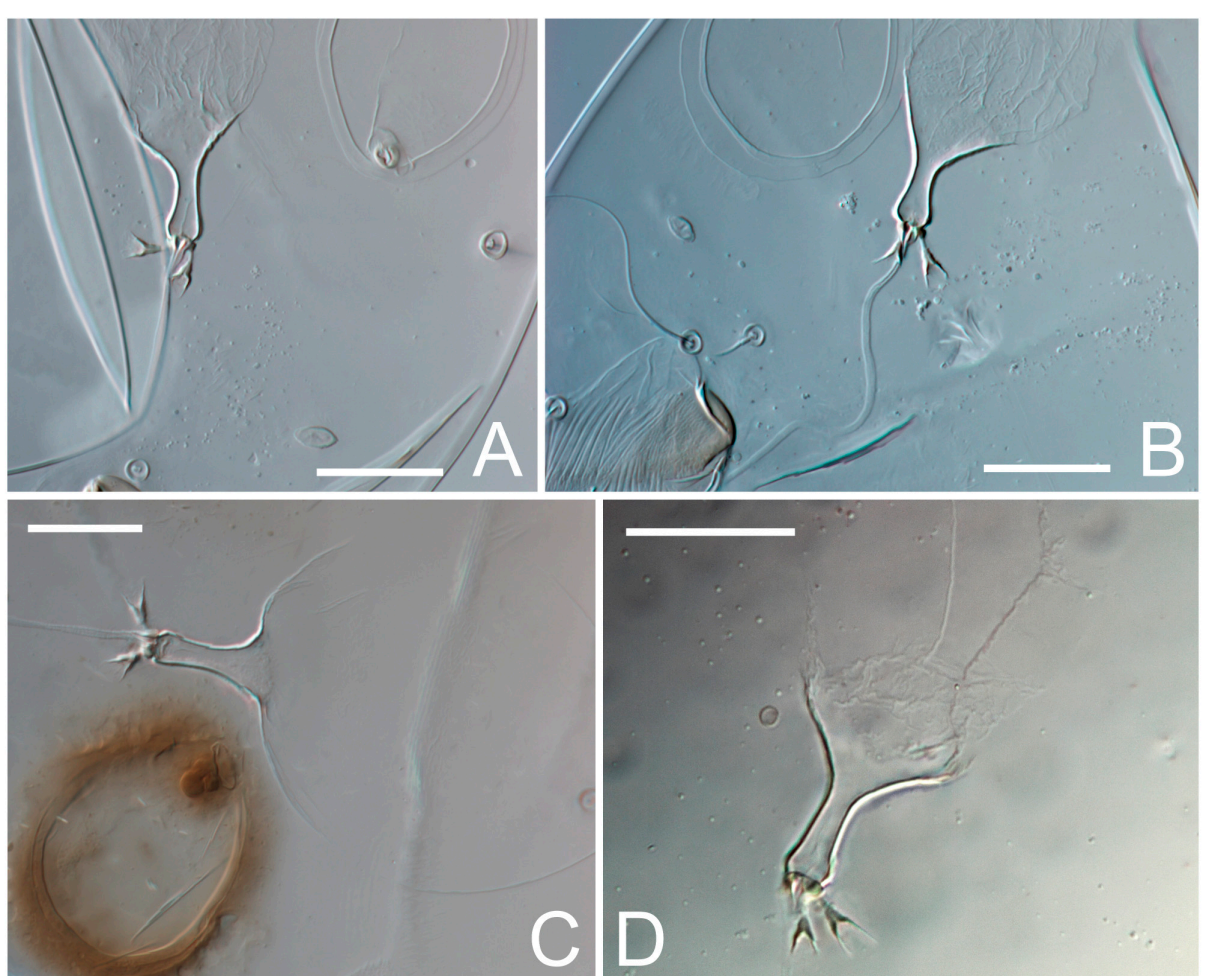
*Thyreophagus tauricus* sp. n., female, paratypes, DIC images: (**A**–**D**)—spermatheca. Scale bar 20 µm.

**Figure 7:**
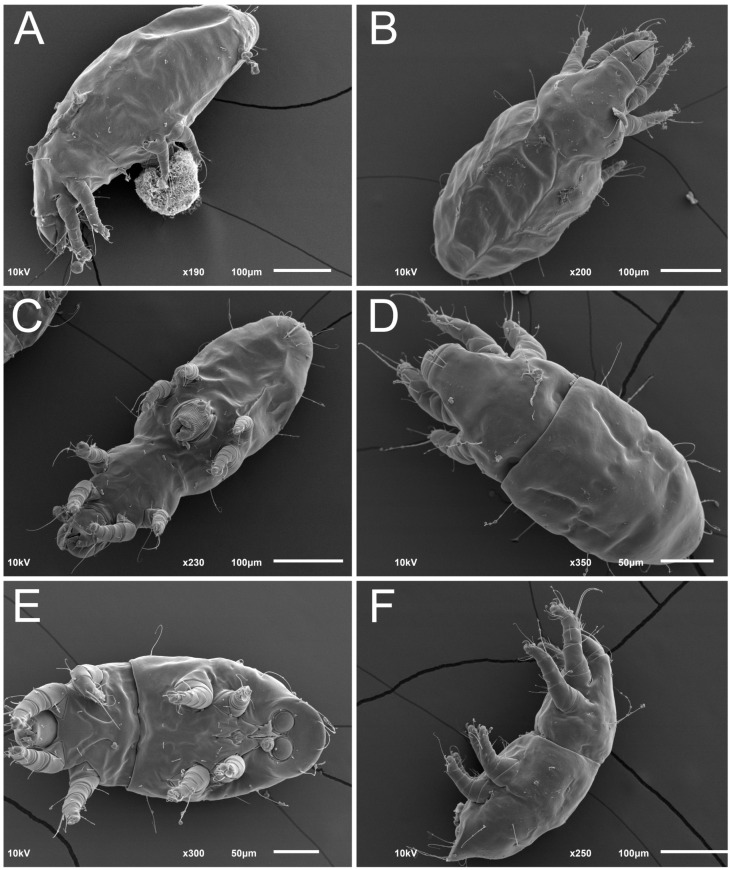
*Thyreophagus tauricus* sp. n., SEM images: (**A**)—female, lateral view; (**B**)—female, dorsal view; (**C**)—female, ventral view; (**D**)—male, dorsal view; (**E**)—male, ventral view; (**F**)—male, lateral view.

**Figure 8:**
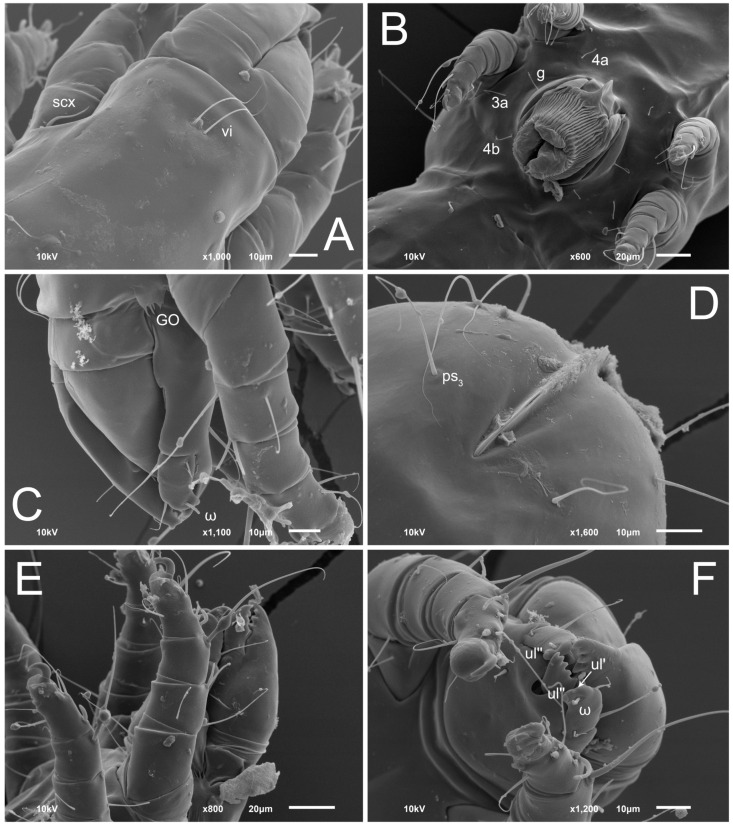
*Thyreophagus tauricus* sp. n., female, SEM images: (**A**)—prodorsal shield, dorsal view; (**B**)—genital area and legs III–IV, ventral view; (**C**,**E**)—gnathosoma and legs I, lateral views; (**D**)—anus, ventral view; (**F**)—gnathosoma and legs I, ventral view.

**Figure 9:**
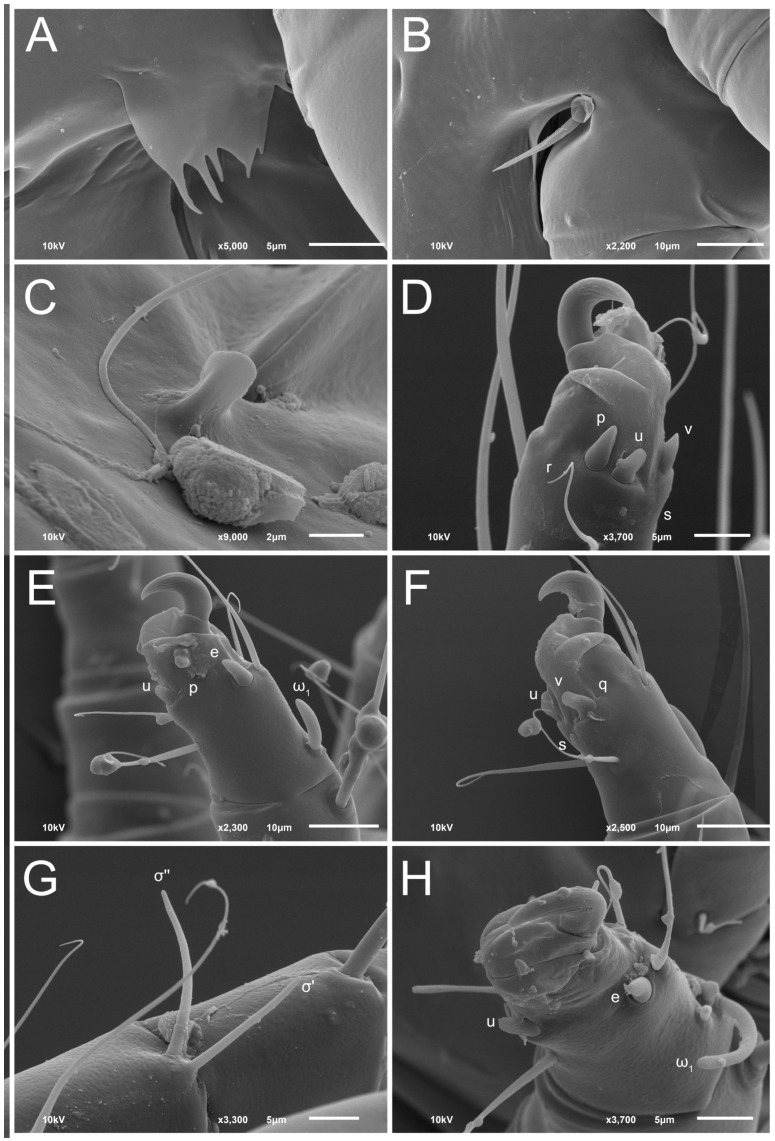
*Thyreophagus tauricus* sp. n., female, SEM images: (**A**)—Grandjean’s organ; (**B**)—supracoxal seta; (**C**)—copulatory tube; (**D**)—tarsus III, posterior view; (**E**)—tarsus II, posterior view; (**F**)—tarsus II, anterior view; (**G**)—solenidion σ I; (**H**)—tarsus I, posterior view.

Female (Figure 1, Figure 2, Figure 3, Figure 4, Figure 5 and Figure 6, Figure 7A–C, Figure 8 and Figure 9). Idiosoma elongate, 500 × 280 (holotype), 400–530 × 190–270 (paratypes, n = 9), 1.8 (2.0–2.1) times longer than wide. Idiosomal cuticle smooth. Subcapitular setae (*h*) long, widened basally; palp tibial setae (*a*), lateral dorsal palp tibial setae (*sup*), dorsal palp tarsal seta (*cm*) filiform; supracoxal seta *elcp* present; terminal palp tarsal solenidion ω short; external part of terminal eupathidium *ul’’* dome-shaped; terminal eupathidium *ul’* small, rounded. Prodorsal sclerite 100 (84–102) long, 97 (75–90) wide, 1.0 (1.1) times longer than wide, with setae *vi* (situated at anterior part of shield, alveoli separated), rounded anterolateral incisions, and elongate midlateral incisions (insertion points of setae *ve*). Prodorsal sclerite smoothly punctate except large lineate central region; posterior end of sclerite with lineate pattern. Grandjean’s organ (GO) with seven membranous finger-shaped processes. Supracoxal setae (*scx*) smooth, sword-shaped, widened and flattened, tapering at tip. Idiosomal setae (*vi*, *se*, *c*_p_, *d*_2_, *e*_2_, *h*_1_, *h*_2_, *h*_3_, *ps*_3_) smooth, filiform and short; opisthosomal gland openings slightly anteriad setal bases *e*_2_. Three pairs of fundamental cupules (*ia*, *im* and *ih*) present, *ip* not observed. Ventral idiosoma with four pairs of coxal setae (*1a*, *3a*, *4a* and *4b*) and 1 pair of genital setae (*g*). Shape of coxal sclerites as in Figure 1B and Figure 4E,F. Genital region situated between coxal fields III and IV; genital valves form an inverted Y; epigynal and medial apodemes well-developed. Diameter of genital papillae approximately 0.3–0.4 the length of coxal and genital setae. Anal opening terminal. Copulatory tube present, situated anterodorsally to anus, with developed opening. Canal of spermatheca long, slender tube-like, leading from copulatory opening to spermatheca, uniformly wide, wider at entrance to spermatheca. Sclerotized vase-shaped atrium of spermatheca with length greater than width, base 3–4 times wider than end of atrium at junction with sclerites of oviducts. Paired Y-shaped sclerites of oviducts, small, elongated.

Legs short, all segments free. Trochanters I–III each with long, filiform seta, *pR* I–II, *sR* III; trochanter IV without setae. Femoral setation 1-1-0-1; setae *vF* I–II and *wF* IV long, filiform. Genual setation 2-2-0-0; setae *mG* and *cG* I–II long, filiform; seta *nG* III absent. Tibial setation 2-2-1-1; setae *hT* I-II represented by alveoli or minute setae; setae *gT* I–II and *kT* III–IV elongate, somewhat spiniform. Tarsal setation 10-10-10-10; pretarsi consists of hooked empodial claws attached to short paired condylophores. Tarsus I and II with setae *ra*, *la*, *f* and *d* filiform, *e*, *u*, *v* spiniform, *p* and *q* represented by small triangular rudiments, *s* flattened, button-shaped or minute spiniform; setae *wa* absent. Tarsus III with setae *f*, *d*, *r* filiform, *e*, *u*, *v*, *p*, *q* spiniform, *s* flattened, button-shaped or minute spiniform, *w* flattened, button-shaped. Tarsus IV similar to tarsus III, except *w* filiform. Solenidion ω_1_ on tarsus I cylindrical, with clavate apex, curved; solenidion ω_1_ on tarsus II simple, cylindrical, with clavate apex, not bent, shorter and wider than ω_1_ on tarsus I. Solenidion ω_2_ on tarsus I shorter than ω_1_, cylindrical, with rounded apex, slightly widened distally, situated slightly distal to ω_1_. Solenidion ω_3_ on tarsus I cylindrical, with rounded tip, subequal to ω_1_, longer than ω_2_. Famulus (ε) of tarsus I wide, spiniform, with broadly rounded apex, widest at middle. Solenidia φ of tibiae I–III elongate, tapering, well extending beyond apices of respective tarsi with ambulacra; solenidion φ IV shorter, shorter than tarsus IV (with ambulacra). Solenidia σ′ and σ″ on genu I elongate, tapering, subequal in length, slightly not reaching bases of φ I. Solenidion σ on genu II more than 6–7 times longer than its width) with rounded tip. Solenidion σ of genu III absent.

**Figure 10:**
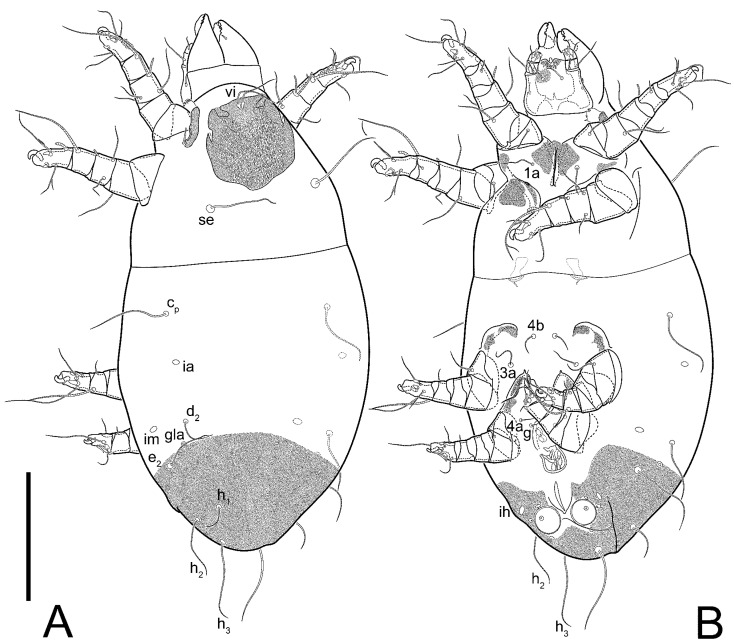
*Thyreophagus tauricus* sp. n., male, paratype: (**A**)—dorsal view; (**B**)—ventral view. Scale bar 100 µm.

**Figure 11:**
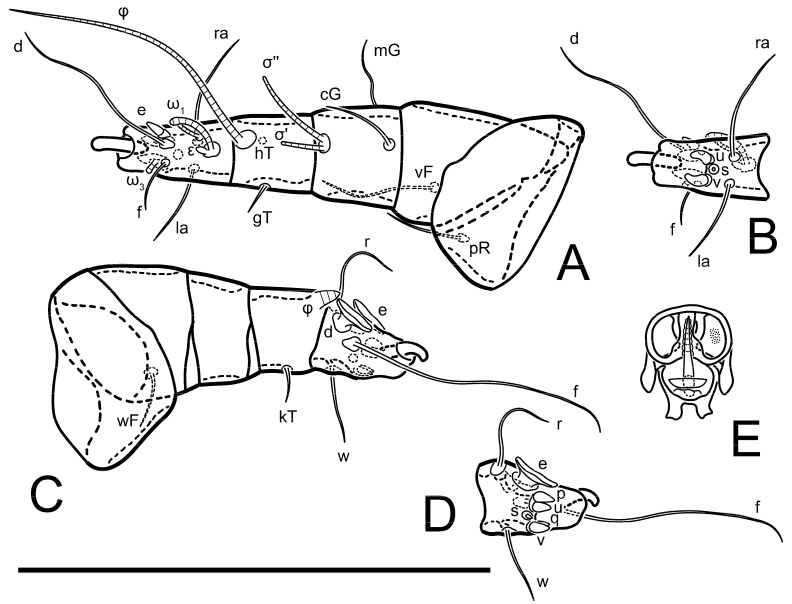
*Thyreophagus tauricus* sp. n., male, paratype: (**A**)—right leg I, dorsal view; (**B**)—tarsus I, ventral view; (**C**)—left leg IV, anterior view; (**D**)—tarsus IV, posterior view; (**E**)—genitalia. Scale bar 100 µm.

**Figure 12:**
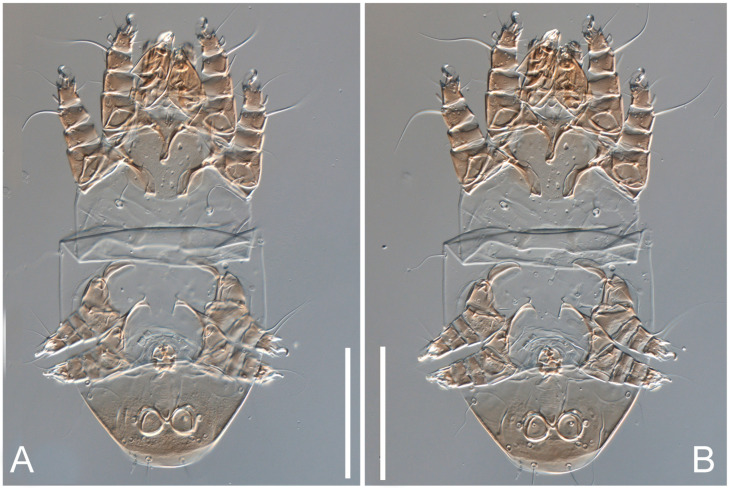
*Thyreophagus tauricus* sp. n., male, paratypes, DIC images: (**A**)—dorsal views, (**B**)—ventral views. Scale bar 100 µm.

**Figure 13:**
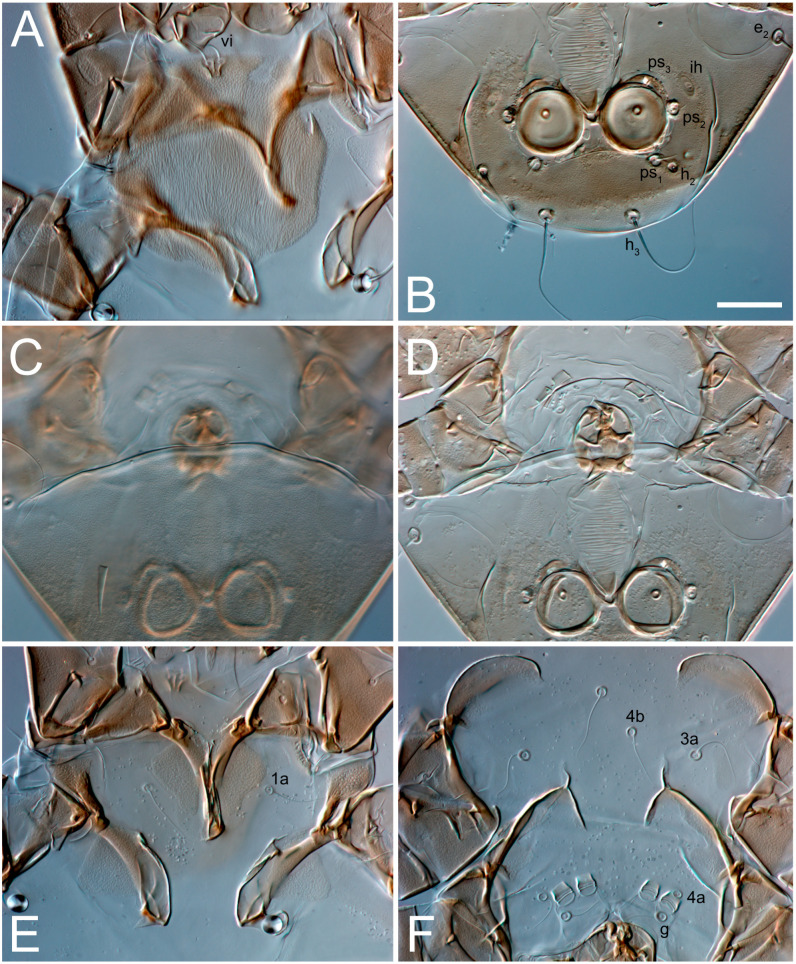
*Thyreophagus tauricus* sp. n., male, paratypes, DIC images: (**A**)—prodorsal shield; (**B**)—anal region, ventral view; (**C**)—opisthonotal shield, dorsal view; (**D**)—genital region, ventral view; (**E**)—coxisternal fields I–II; (**F**)—coxisternal fields III–IV. Scale bar 20 µm.

**Figure 14:**
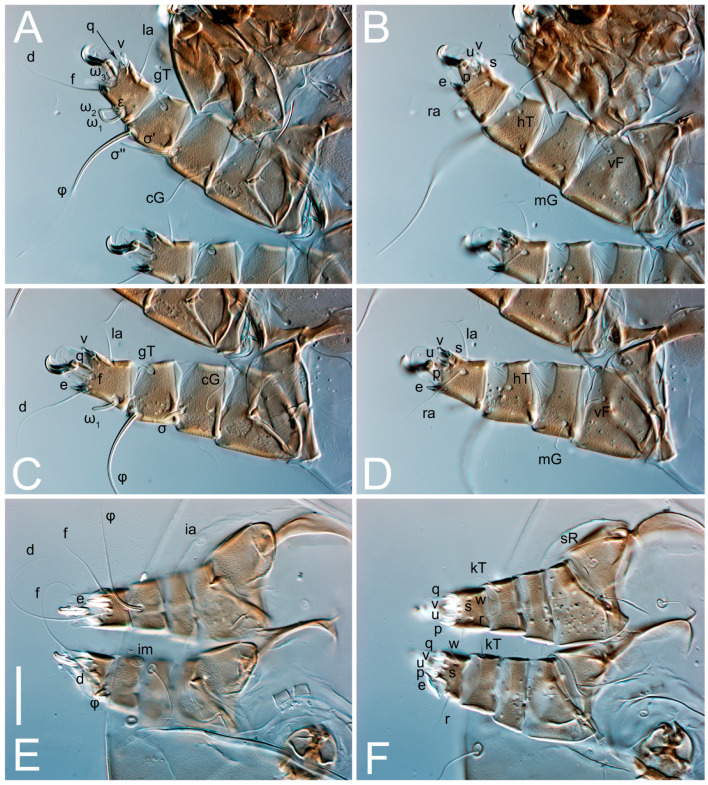
*Thyreophagus tauricus* sp. n., male, paratypes, DIC images: (**A**)—left leg I, anterior view; (**B**)—left leg I, posterior view; (**C**)—left leg II, anterior view; (**D**)—left leg II, posterior view; (**E**)—left legs III–IV, dorsal view; (**F**)—left legs III–IV, ventral view. Scale bar 20 µm.

**Figure 15:**
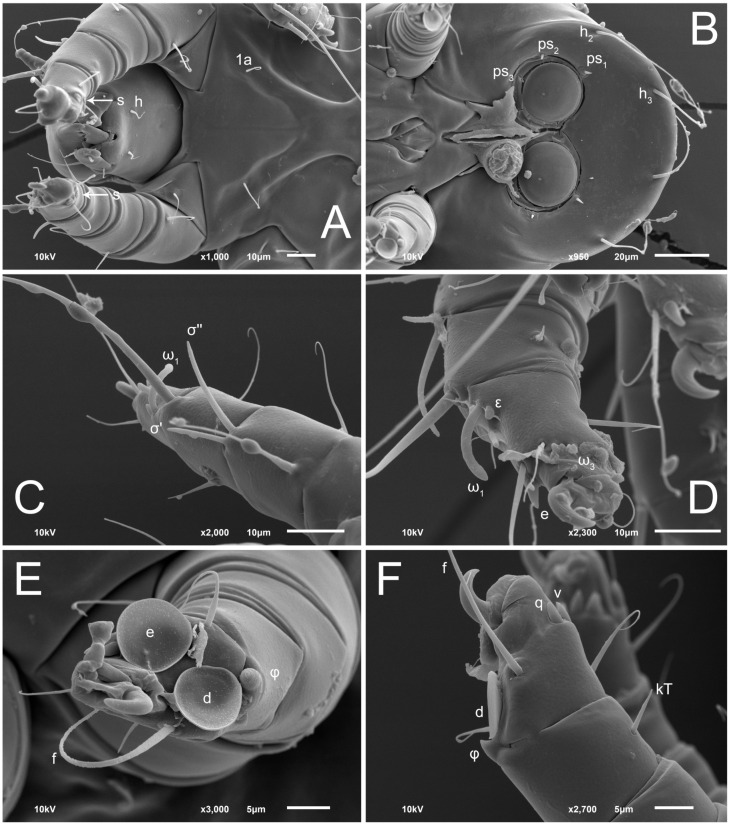
*Thyreophagus tauricus* sp. n., male, SEM images: (**A**)—gnathosoma, legs I and coxisternal fields I, ventral view; (**B**)—anogenital area, ventral view; (**C**)—tibia and tarsus I, dorsal view; (**D**)—tarsus I, anterior view; (**E**)—leg IV, dorsal view; (**F**)—tarsus IV, anterior view.

Male (n = 4, paratypes). (Figure 7D–F and Figure 10, Figure 11, Figure 12, Figure 13, Figure 14 and Figure 15). Idiosoma elongate, 300–400 × 160–210, 1.9–2.1 times longer than wide. Idiosomal cuticle smooth. Gnathosoma as in female. Prodorsal sclerite 63–78 long, 58–73 wide, 1.1–1.2 times longer than wide, with setae *vi*, incisions and ornamented as in female. Grandjean’s organ (GO) with 5–7 membranous processes. Supracoxal seta (*scx*) as in female. Idiosomal setae (*vi*, *se*, *c*_p_, *d*_2_, *e*_2_, *h*_1_, *h*_2_, *h*_3_) smooth, filiform and short; opisthosomal gland openings slightly anteriad setal bases *e*_2_. Three pairs of fundamental cupules (*ia*, *im* and *ih*) present, *ip* not observed. Opisthonotal shield smoothly punctate; ventral part extends to anal suckers. Ventral idiosoma with four pairs of coxal setae (*1a*, *3a*, *4a* and *4b*) and one pair of genital setae (*g*). Shape of coxal sclerites on Figure 10B and Figure 13E,F. Genital region between coxisternal fields IV; arms of genital capsule rounded; aedeagus short, not protruding beyond anterior edge of genital capsule. Diameter of genital papillae approximately 0.3–0.4 the length of coxal and genital setae. Anal suckers rounded in outline. Setae *ps*_1–3_ very short.

Legs I-III as female, except solenidion ω_3_ on tarsus I very short, truncated; and solenidion σ″ about two-times longer than σ′. Trochanter and genu IV without setae, femur IV with *wF* IV long, filiform, tibia IV with *kT* IV elongate, somewhat spiniform. Tarsus IV with 10 setae, of them, *f*, *r*, *w* filiform, *d* and *e* represented by suckers, *u*, *v*, *p*, *q* spiniform, *s* flattened, button-shaped or minute spiniform. Solenidion φ on tibia IV short and wide.

**Figure 16:**
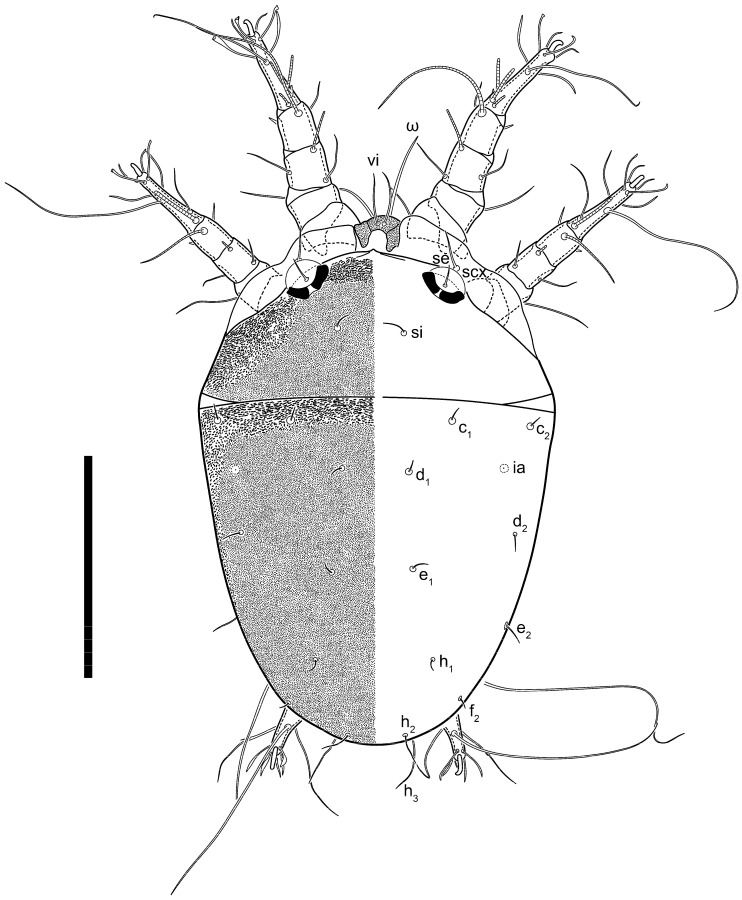
*Thyreophagus tauricus* sp. n., phoretic deutonymph, paratype, dorsal view. Scale bar 100 µm.

**Figure 17:**
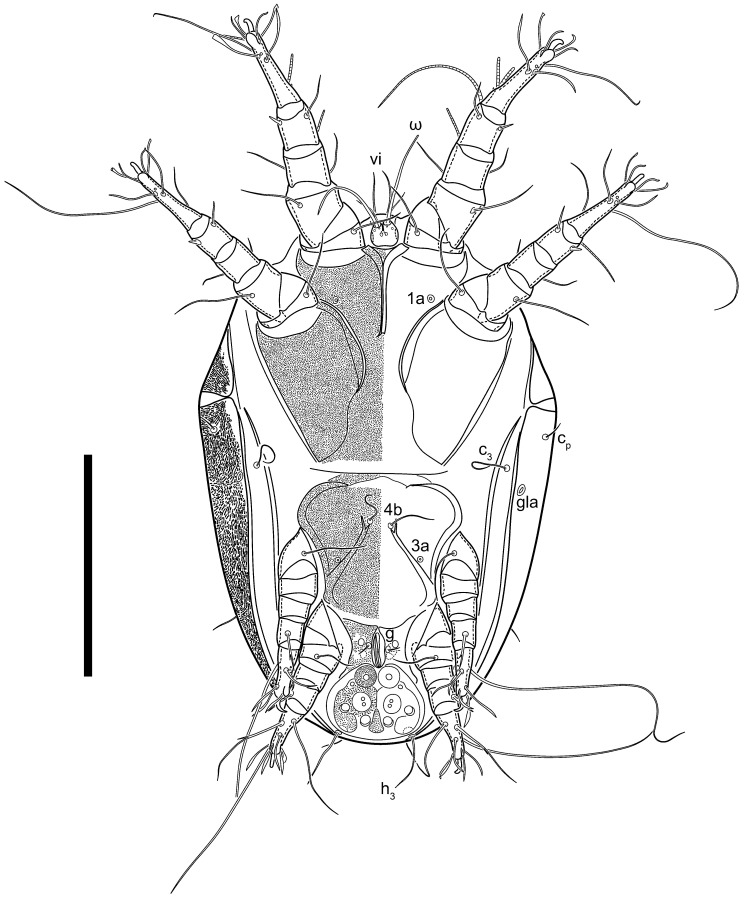
*Thyreophagus tauricus* sp. n., phoretic deutonymph, paratype, ventral view. Scale bar 100 µm.

**Figure 18:**
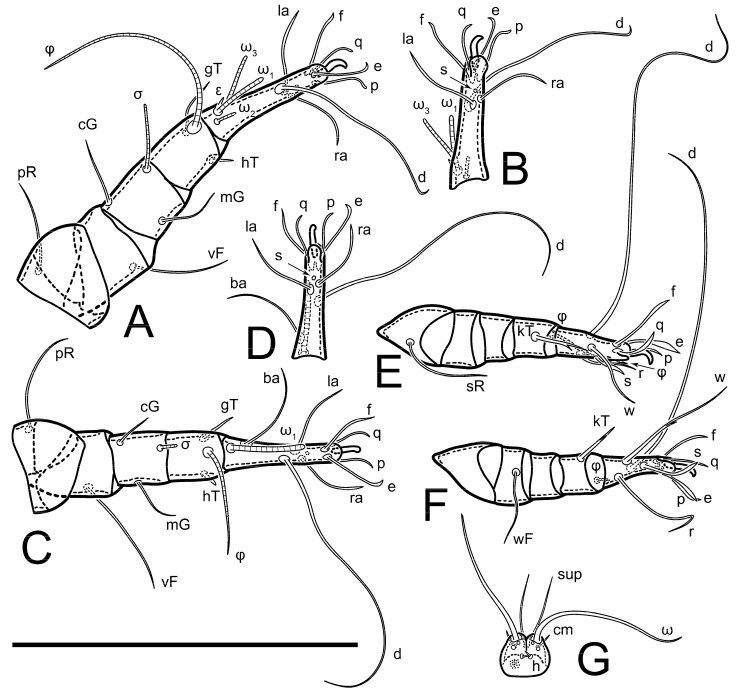
*Thyreophagus tauricus* sp. n., phoretic deutonymph, paratype: (**A**)—right leg I, posterior view; (**B**)—tarsus I, anterior view; (**C**)—right leg II, dorsal view; (**D**)—tarsus II, ventral view; (**E**)—right leg III, anterior view; (**F**)—right leg IV, ventral view; (**G**)—gnathosoma, ventral view. Scale bar 100 µm.

**Figure 19:**
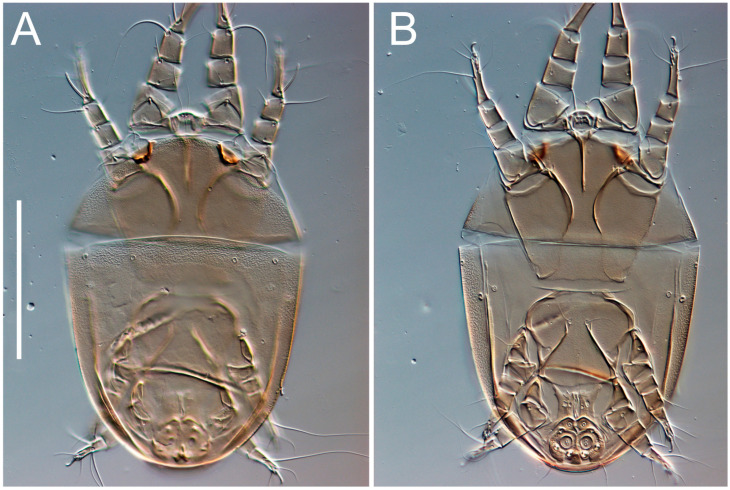
*Thyreophagus tauricus* sp. n., phoretic deutonymph, paratype, DIC images: (**A**)—dorsal view; (**B**)—ventral view. Scale bar 100 µm.

**Figure 20:**
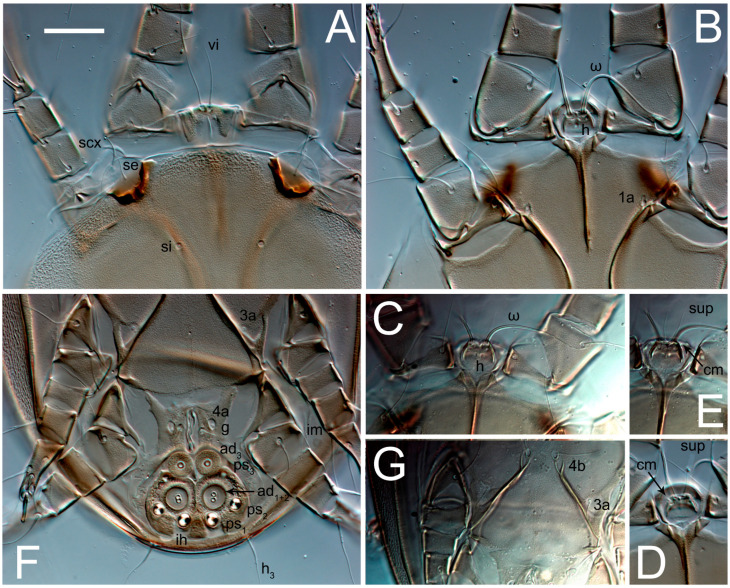
*Thyreophagus tauricus* sp. n., phoretic deutonymph, paratype, DIC images: (**A**)—prodorsum; (**B**)—propodosoma, ventral view; (**C**)—gnathosoma, ventral view; (**D**,**E**)—gnathosoma, dorsal views; (**F**,**G**)—hysterosoma, part, ventral view. Scale bar 20 µm.

**Figure 21:**
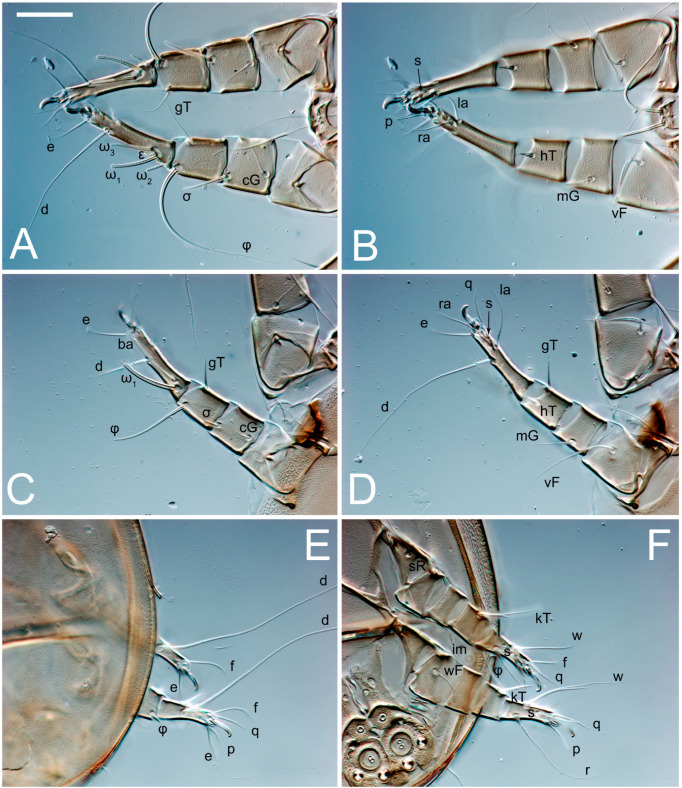
*Thyreophagus tauricus* sp. n., phoretic deutonymph, paratype, DIC images: (**A**)—legs I, dorsal view; (**B**)—legs I, ventral view; (**C**)—left leg II, dorsal view; (**D**)—left leg II, ventral view; (**E**)—right legs III–IV, dorsal view; (**F**)—right legs III–IV, ventral view. Scale bar 20 µm.

Phoretic deutonymph (n = 4, paratypes) (Figure 16, Figure 17, Figure 18, Figure 19, Figure 20 and Figure 21). Body elongate, 1.33–1.44 times longer than wide, widest in sejugal region; idiosomal length 220–240 width 153–180. Gnathosoma short, subcapitulum and palps fused, bearing palpal solenidia (ω) apically and filiform apicodorsal setae (*sup*); setae *h* present, minute (Figure 20C) or absent (their positions marked by somewhat refractile spots), setae *cm* present.

Dorsum. Idiosoma smoothly punctate; distinct linear pattern present on anterior and lateral sides of prodorsal sclerite and hysterosomal shield. Apex of propodosoma anterior to anterior border of prodorsal sclerite, with apical internal vertical setae (*vi*) (bases separated) and a pair of band-like sclerites coalescing anteriorly. A pair of lateral, widely separated ocelli (distance 40–49) present on prodorsum; lenses and pigmented spots present; maximal diameter of lenses 18–20. External vertical setae (*ve*) absent; external scapular setae *se* situated below lenses; internal scapular setae (*si*) distinctly posterior and medial to external scapulars (*se*). Supracoxal setae of legs I (*scx*) filiform, with extended base, positioned below *si* and anterolaterad to ocelli. Sejugal furrow well developed. Prodorsal sclerite 67–73, hysterosomal shield 150–160, ratio hysterosoma shield/prodorsal sclerite length = 2.2–2.3. Hysterosoma with 11 pairs of simple, filiform setae on hysterosomal shield (*c*_1_, *c*_2_, *c*_p_, *d*_1_, *d*_2_, *e*_1_, *e*_2_, *f*_2_, *h*_1_, *h*_2_, *h*_3_), setae *h*_3_ distinctly longer than others. Opisthonotal gland openings (*gla*) situated ventrally on hysterosomal shield, slightly posterior to setae *c*_3_. Of four fundamental pairs of cupules, three pairs were observed: *ia* posteriomediad setae *c*_2_, *im* ventral, laterad of trochanters IV and *ih* ventral, laterad posterior sides of attachment organ.

Venter. Coxal fields sclerotized, smoothly punctate. Anterior apodemes of coxal fields I fused forming sternum; sternum not reaching posterior border of sternal shield by distance exceeding its length. Posterior border of sternal shield not sclerotized. Anterior apodemes of coxal fields II curved medially. Posterior apodemes of coxal fields II weakly developed, thin, curved medially. Sternal and ventral shield contiguous. Anterior apodemes of coxisternal fields III free, connected by thin transverse sclerotization. Posterior medial apodeme in area of coxisternal fields IV weakly developed. Posterior apodemes IV absent. Subhumeral setae (*c*_3_) filiform, situated on ventral surface between legs II–III, adjacent to region separating sternal and ventral shields. Coxal setae *1a*, *3a* reduced, represented by minute structures or filiform, situated in alveoli (Figure 20G). Setae *4b*, *g* filiform; *4a* in form of small, rounded conoids. Genital region in posterior portion of coxisternal fields IV; opening elongate, with two pairs of genital papillae within genital atrium; papillae two-segmented, with rounded apices. Coxal setae (*4b*) situated at tips anterior coxal apodemes IV; genital setae (*g*) laterad of genital opening. Attachment organ posterior to coxisternal fields IV. Anterior suckers (*ad*_3_) round, median suckers (*ad*_1+2_) distinctly larger, with paired vestigial alveoli (not situated on common sclerite); pair of small refractile spots anterolateral to median suckers (*ps*_3_); lateral conoidal setae of attachment organ (*ps*_2_) situated distinctly posterior to line joining centers of median suckers, slightly anterior to conoidal setae (*ps*_1_); anterior and posterior lateral and posterior median cuticular conoids well developed; anus situated between anterior suckers (*ad*_3_).

Legs. Legs elongate, all segments free. Trochanters I–III each with long, filiform seta, *pR* I–II, *sR* III. Femoral setation 1-1-0-1; setae *vF* I–II and *wF* IV long, filiform. Genual setation 2-2-0-0; setae *mG* and *cG* I–II filiform, seta *nG* III absent. Tibial setation 2-2-1-1; setae *hT* I somewhat spiniform; setae *gT* I filiform; setae *gT* and *hT* II spiniform, setae *gT* II longer than *hT* II; setae *kT* III spiniform and with distinct prong; setae *kT* IV somewhat spiniform, shorter than *kT* III, with short prong. Tarsal setation 8-9-8-8. All pretarsi consisting of hooked empodial claws attached to short paired condylophores. Tarsus I with setae *ra*, *la*, *p*, *q*, *d*, *f* narrowly lineolate; *e* slightly spoon-shaped; seta *d* elongate, its base at level of *ra* and *la*; seta *s* represented by alveolus; setae *wa*, *aa* and *ba* I absent; tarsus II similar to tarsus I except seta *ba* present, filiform, situated close to ω_1_. Tarsus III with setae *w*, *r*, *s*, *p*, *q*, *e*, *f* and *d* smooth; all setae, except for *d* III, more or less foliate; seta *d* longer than leg. Tarsus IV similar to tarsus III, except seta *r* longer, filiform; seta *w* filiform and with distinct prong. Solenidia ω_1_ on tarsi I–II cylindrical, with slightly clavate apices; ω_3_ on tarsus I slightly shorter than ω_1_, with rounded apex, situated slightly distal to ω_1_; ω_1_ and ω_3_ separated by bulbous famulus (ε); solenidion ω_2_ of tarsus I expanding slightly apically, situated somewhat more basal and posterior to ω_1_ + ε + ω_3_ group; solenidia φ of tibiae I–III elongate, tapering; φ I longer than tarsus I; φ II shorter than tarsus II; φ III reaching tip of tarsus III without ambulacrum; φ IV short; σ of genu I elongate, slightly tapering, nearly reaching tip of tibia I; σ of genu II much shorter, cylindrical, not reaching midlength of tibia II; σ of genu III absent.

Type material. Holotype (female) and paratypes (14 females, 9 males and 4 heteromorphic deutonymphs) from lab culture; culture started from specimens collected in Crimea, vicinity of Yalta, under the bark of fallen twigs of *Tilia* sp., 6 April 2022, 44.483333 N, 34.083333 E, coll. Khaustov V.A, PBK 20-0101-065.

Depository. The holotype and paratypes (11 females, 6 males and 1 heteromorphic deutonymphs) are deposited in the Museum of Zoology, Tyumen State University, Russia. The remaining paratypes (4 females, 3 males and 3 heteromorphic deutonymphs) are deposited in the Zoological Institute, Russian Academy of Sciences, Saint Petersburg, Russia.

Etymology. Tauricus (of Taurica, Lat. adjective). Taurica is a historical name of the Crimean Peninsula used by the Greeks and Romans. This is a nomen in supposition.

Diagnosis. Adults of *Thyreophagus tauricus* are distinct among all other species of the genus by the presence of flattened, button-shaped or minute spiniform (much less than *v* and *p*) setae *s* III-IV (Figure 9F) (vs. well-developed spiniform (not much less than *v* and *p*) in all other species, the adults of which are known). The new species is close to *Th. corticalis* (patterns of prodorsal sclerite, length of dorsal setae and shaped of legs setae, except *s* III–IV), but differs from it in having the vase-shaped atrium of the spermatheca, which is 3–4 times wider at the basal part than at junction with sclerites of oviducts (vs. 1.5–2 times wider in *Th. corticalis*).

Heteromorphic deutonymphs of *Th. tauricus* are distinct from all other species of *Thyreophagus* by the presence of the well-developed setae *cm* on the dorsal part of the subcapitular remnant (vs. absent all other species). *Thyreophagus tauricus* has a prong on *kT* III (as in *Th. australis* Clark, 2009 and *Th. sminthurus* (Fain and Johnston, 1974)), but it differs from *Th. australis* in having the larger ocelli 20 (vs. 10 in *Th. australis*). *Thyreophagus tauricus* differs from *Th. sminthurus* in having the wider idiosoma, which is 1.33–1.44 times longer than wide (vs. 2.1 in *T. sminthurus*), and the presence of tibial setae *hT* I, II (vs. absent in *Th. sminthurus*).

## 4. Discussion

In recent years, there has been a growing interest in finding sustainable and environmentally friendly solutions to combat agricultural pests while minimizing the adverse impacts of chemical pesticides. Mite species of the genus *Thyreophagus* represent an exciting alternative to synthetic pesticides in the realm of pest control in agriculture. For example, *Thyreophagus entomophagus* and *Thyreophagus calusorum* are widely used as factitious prey for mass rearing of phytoseiid predatory mites for biocontrol applications [9,15], while *Thyreophagus cracentiseta*, has been proposed as such a species based on its performance in laboratory experiments [7]. As several species of *Thyreophagus* have proven to be valuable factitious prey for the mass rearing of predatory mites, these predatory mites, in turn, play a crucial role in biological pest control [26]. They feed on a variety of agricultural pests such as spider mites, thrips and other small arthropods that harm crops [27]. The significance of this approach lies in its potential to reduce our reliance on synthetic pesticides. Unlike chemical pesticides, *Thyreophagus*-based biocontrol methods are environmentally sustainable, as they do not introduce harmful chemicals into the ecosystem. This approach is highly targeted, focusing solely on the pests without affecting beneficial organisms or pollinators. It is also adaptable to various crops and integrated pest management systems [26]. Furthermore, the use of *Thyreophagus* mites to produce predatory mites for biocontrol can potentially lead to a reduction in pesticide residues on agricultural products, making them safer for consumption. As the demand for organic and environmentally friendly farming practices continues to grow, the role of *Thyreophagus* mites in replacing synthetic pesticides becomes increasingly significant, offering a promising and sustainable solution for pest management in agriculture.

Given the economic significance of *Thyreophagus*, it is important to know its biodiversity, habitat and species boundaries based both on morphology and DNA sequences. However, these aspects remain significantly underexplored. For instance, it is noteworthy that a substantial number of *Thyreophagus* species are thought to be yet undescribed [1,8] even in regions where extensive biodiversity research has been carried out, notably in Europe and North America [4]. In addition, there is only a single sequence of *Thyreophagus* “*entomophagus*” in GenBank; however, this sequence is likely based on misidentification (see below). The prevalence of undescribed or poorly characterized species raises important questions about our understanding of the global *Thyreophagus* biodiversity. Numerous *Thyreophagus* mite species live in subcortical environments, alongside scale insects, or within the nests of bees and wasps, thus eluding their discovery due to their secretive lifestyles and cryptic habitats. Understanding and documenting these species is crucial for achieving a comprehensive picture of the global *Thyreophagus* biodiversity. Curiously, as *Thyreophagus* mites are adapted to live in concealed habitats, which have limited number of natural predators, this adaptation is one of the biological features that render *Thyreophagus* mites useful as factitious prey in industrial settings. These mites, characterized by slow movement and a lack of many natural defenses, such as long setae, are particularly suitable as prey for phytoseiid mites when reared industrially. Therefore, as *Thyreophagus* mites play an important role in biocontrol applications, a more thorough examination of their diversity and biology is also essential. Improved knowledge of these mites would not only facilitate their use in pest management but also potentially uncover new species suitable for local production of phytoseiid mites, thereby minimizing the risk of introducing non-native *Thyreophagus* species into new regions. In light of these considerations, it is clear that a more concerted effort is needed to study *Thyreophagus* mites comprehensively. This entails employing a range of research methods, from intensive fieldwork to laboratory-based studies. Additionally, molecular techniques can aid in precisely identifying and classifying these mites, shedding light on their genetic diversity and evolution.

Here, we report the discovery of a new sexual species, *Thyreophagus tauricus* sp. n., and provide thorough analyses of its morphology, key life stages, molecular characteristics, and its relationship with other species. As we established a pure culture of this new species in the laboratory, we were able to confidently correlate all its taxonomically important life stages, males, females and deutonymphs (a dispersal stage). To facilitate molecular identification and species delimitation, we sequenced the entire COX1 gene, a useful DNA barcoding gene [28] of four Palearctic species: *Th. tauricus*, *Th. corticalis*, *Th. calusorum* and *Th. entomophagus*. Of them, the two latter species are used for mass-rearing of phytoseiid mites [9,15]. We found that the new species is morphologically close to *Th. corticalis*, a widely distributed Palearctic species; however, it differs from *Th. corticalis* and other *Thyreophagus* species by the following character states: adults of *Th. tauricus* are distinct by the presence of flattened, button-shaped or minute spiniform setae *s* III-IV, which are well-developed and spiniform in all other known species of *Thyreophagus*; heteromorphic deutonymphs of *Th. tauricus* are distinct from all other species of *Thyreophagus* by the presence of well-developed setae *cm* on the dorsal part of the subcapitular remnant (absent in all other species). Despite being very close to *Th. corticalis*, genetic COX1 K2P distances were large, 19.8% (Table 1), suggesting the presence of a well-delimited species, *Th. tauricus* sp. n., which is distant by both morphology and DNA sequences. COX1 K2P distances between *Th. tauricus* and other species (*Th. entomophagus*, *Th.* “*entomophagus*” NC 066986.1, *Th. calusorum*, *Th. corticalis*) ranged between 20.1 and 24.3% (Table 1). 

As part of our study, we also verified sequences deposited into the GenBank database. One such sequence from China (NC 066986.1) was initially identified as ‘*Th. entomophagus*’. However, our sequence, derived from a pure, industrially produced European culture carefully identified by us, displayed a significant 21.16% COX1 K2P distance from this GenBank sequence (Table 1). This substantial genetic distance strongly suggests that the GenBank sequence NC 066986.1 was misidentified and does not belong to *Th. entomophagus*. This raises questions about the reliability of public databases and the importance of rigorous verification and validation in biological research.

## 5. Conclusions

The discovery and comprehensive study of *Thyreophagus tauricus* underscore the importance of biodiversity research, taxonomic methods, molecular techniques and the need for rigorous scientific practices. Understanding the diversity and characteristics of mite species like *Thyreophagus* can have broader implications for ecology, agriculture and biocontrol efforts.

## Figures and Tables

**Table 1:** COX1 nucleotide distances of four species of the genus *Thyreophagus*. Uncorrected p-distances are in the upper diagonal, K2P distances are in the lower diagonal.

	*Th. “entomophagus*”	*Th. calusorum*	*Th. entomophagus*	*Th. corticalis*	*Th. tauricus*
*Th. “entomophagus*” China NC 066986.1	-	0.1686	0.1834	0.1699	0.2059
*Th. calusorum* OR640973	0.1929	-	0.1705	0.1705	0.1905
*Th. entomophagus* OR640974	0.2116	0.1942	-	0.1538	0.1750
*Th. corticalis* OR640975	0.1934	0.1984	0.1732	-	0.1725
*Th. tauricus* OR640976	0.2428	0.2216	0.2008	0.1980	-

## Data Availability

Data are available in a publicly accessible repository.
